# The Cozy Fireside

**Published:** 1874-12

**Authors:** 


### THE COZY FIRESIDE.

In every home there should be a room the cheeriest and best in all the house, where all the members of the family should oftenest meet together, and to which everyone might feel free to go at all times of the day or night. To make this room attractive and delightful should be the aim and effort of every member of the household, by the cultivation of cheerful courtesies and the constant practice of all the social amenities; in every way endeavoring to promote mutual pleasure, profit and enjoyment. The winter night gathers in; the shutters are closed; the blinds drawn down; the winds whistle dolefully on the outside; the cold rain patters on the lattice; but in the favored room, there is the mother at her needle; father is reading the evening newspaper; the larger children are gathered around the centre table, learning their lessons for the morrow, while the smaller ones are tumbling over the floor in merry play. There are the old shovel and tongs, and there, too, is "pussy cat," doubled up in a coil on the hearth rug, or on fore feet erect looks steadily——at the register or fire; which? That makes all the difference in the world. There may be glittering candelabras, with their dozen of lights; there may be blazing gas jets all around the room; paintings of rare value and beauty may adorn the walls; papers and magazines may cover the table, while the latest books, in choicest bindings, fill the library shelves; but who does not know that a brightly blazing fire on the hearth intensifies the charm of all these charming things, and excites a mental exhilaration which deepens every enjoyment and adds to every pleasure.

Every one feels and knows that an open, cheerful, blazing fire on the hearth, whether of wood or coke or coal, imparts a life to a family circle of a cold winter night which nothing else can; while all scientific men admit, most unhesitatingly, that for effectual ventilation and healthfulness no mode of heating an apartment can compare with an open fire. It is very certain that any large family, in city or country, favored with such a "sitting room," and spending most of their time in it, instead of in small apartments warmed, through registers, with furnace heat, with windows hermetically sealed with pestiferous weather strips, there would be a saving in doctors bills, to say nothing of bodily suffering, which would agreeably surprise an economical and thrifty housekeeper. The old-fashioned wood fire, with back log and fore stick, and irons and tongs, are the perfection of coziness and comfort; but wood is too expensive. The next best and most available for many years was the modern "grate;" but the Dixon low down grate gives literally "a fire on the hearth" of the olden time, which is made with wood coke, or soft or hard coal, with no increased expenditure of fuel over that of a grate or furnace.

There are many days from November to May which are so soft or warm that the kindling of a fire in a furnace makes the atmosphere of the whole house so heavy and so oppressive that comfort is impossible. Then there are many other days in which but little fire is needed, yet the servants will persist in building fires appropriate to a zero temperature. Besides all this, in the spring and fall, a blazing fire in the morning and at sundown would be a great comfort to the whole household, while none at all is needed for the middle of the day; in all such cases a few sticks of wood or a few lumps of Liverpool or cannel coal could be used to burn for an hour or two. The prejudice of custom is sometimes both curious and amusing. New York was alive to the advantages of street cars a quarter of a century almost before Philadelphia could be persuaded to even try the experiment; then, in a very short time, the whole city was grid-ironed with tramways. On the other hand, there are thousands of low-down grates in Philadelphia to-day, especially among professional and educated men, and perhaps not a dozen in New York city, although for their convenience, healthfulness and economy there ought to be at least one in every dwelling.

